# Risk Prediction of Second Primary Malignancies in Primary Early-Stage Ovarian Cancer Survivors: A SEER-Based National Population-Based Cohort Study

**DOI:** 10.3389/fonc.2022.875489

**Published:** 2022-05-19

**Authors:** Jiaqin Xu, Chen Huang, Zhenyu Wu, Huilin Xu, Jiong Li, Yuntao Chen, Ce Wang, Jingjing Zhu, Guoyou Qin, Xueying Zheng, Yongfu Yu

**Affiliations:** ^1^ Department of Biostatistics, School of Public Health, Fudan University, Shanghai, China; ^2^ Shanghai Minhang Center for Disease Control and Prevention, Shanghai, China; ^3^ Department of Clinical Epidemiology, Aarhus University, Aarhus, Denmark; ^4^ Department of Epidemiology and Public Health, University College London, London, United Kingdom; ^5^ Shanghai Institute of Infectious Disease and Biosecurity, Shanghai, China

**Keywords:** ovarian cancer, second primary malignancies, competing risk model, nomogram, SEER database

## Abstract

**Purpose:**

This study aimed to characterize the clinical features of early-stage ovarian cancer (OC) survivors with second primary malignancies (SPMs) and provided a prediction tool for individualized risk of developing SPMs.

**Methods:**

Data were obtained from the Surveillance, Epidemiology and End Results (SEER) database during 1998–2013. Considering non-SPM death as a competing event, the Fine and Gray model and the corresponding nomogram were used to identify the risk factors for SPMs and predict the SPM probabilities after the initial OC diagnosis. The decision curve analysis (DCA) was performed to evaluate the clinical utility of our proposed model.

**Results:**

A total of 14,314 qualified patients were enrolled. The diagnosis rate and the cumulative incidence of SPMs were 7.9% and 13.6% [95% confidence interval (CI) = 13.5% to 13.6%], respectively, during the median follow-up of 8.6 years. The multivariable competing risk analysis suggested that older age at initial cancer diagnosis, white race, epithelial histologic subtypes of OC (serous, endometrioid, mucinous, and Brenner tumor), number of lymph nodes examined (<12), and radiotherapy were significantly associated with an elevated SPM risk. The DCA revealed that the net benefit obtained by our proposed model was higher than the all-screening or no-screening scenarios within a wide range of risk thresholds (1% to 23%).

**Conclusion:**

The competing risk nomogram can be potentially helpful for assisting physicians in identifying patients with different risks of SPMs and scheduling risk-adapted clinical management. More comprehensive data on treatment regimens and patient characteristics may help improve the predictability of the risk model for SPMs.

## Introduction

Ovarian cancer (OC) is the third most common tumor among gynecological malignancies with an estimated 21,410 new cases diagnosed in the United States in 2021 (1). Attributed to the advancements in tumor early detection approaches and efforts of screening programs, more patients are being diagnosed at an early stage (stages I–II), resulting in an increasing number of tumor survivors (2). The risk of second primary malignancies (SPMs) among those survivors is becoming a major concern for survival rather than the initial malignancy (3). An investigation of the characteristics and risk of SPMs in cancer survivors may help in the screening and early diagnosis of SPMs.

Previous studies have found that OC survivors have a higher risk of SPMs than the general population (4–7), especially those with early-stage disease (7). However, the characteristics and risk factors for SPMs in OC patients have not been well studied. A case–control study based on 2 oncology hospitals in Europe and 11 population-based cancer registries in Canada and Europe found that the risk of subsequent leukemia was associated with different treatment regimens (8). A Swedish register-based study indicated that a family history of particular cancer contributed to an elevated risk of SPMs at the same site for OC patients (9). Using the Cox proportion hazard model, a study in Taiwan reported that older age, chemotherapy, and radiotherapy were risk factors for SPMs in patients with OC (6). However, these studies mainly focused on a specific risk factor, or did not consider non-SPM death as a competing event for SPMs, which was subject to a biased estimate of cumulative incidence (10). Additionally, a system that can quantitatively estimate the probability of developing SPMs for OC individuals has not been established yet, especially in early-stage patients, for whom routine monitoring and screening of SPMs may yield greater health economic benefits compared with metastatic OC patients.

We carried out a population-based cohort using data from the Surveillance, Epidemiology, and End Results (SEER) database. Using the Fine and Gray model with consideration of competing events, our study aimed to (1) estimate the cumulative incidence of SPMs in early-stage OC survivors; (2) explore related risk factors of SPMs; and (3) construct a competing-risk nomogram to predict individual 3-, 5-, and 10-year probabilities of SPMs.

## Materials and Methods

### Data Source

Data were obtained from the SEER program of the National Cancer Institute, which collected data from 18 population-based cancer registries that covered approximately 28% of the total United States population (11). The SEER registries collected information on patient demographics, tumor characteristics, treatment methods, and follow-up survival data complying with the strict data-quality indicators. Thus, the SEER database is the largest cancer database worldwide and the most authoritative source of cancer statistics in the US.

### Study Population

The patients were identified according to the 3rd edition of the International Classification of Disease for Oncology (ICD-O-3/WHO 2008), and the initial cancer site was restricted to “Ovary”. The last follow-up date for the latest SEER data was 31 December 2018, and the treatment information on whether to receive surgery, chemotherapy, and radiotherapy was only available since 1998. Therefore, we included patients diagnosed between 1 January 1998 and 31 December 2013 to ensure at least a 5-year follow-up to observe the risk of developing SPMs. The tumor stage was recoded according to the 8th edition of the AJCC cancer staging manual (12) based on primary tumor, regional node, and distant metastasis stages (**Supplementary Table 1**). The patients whose lesions outside the pelvis or with distant metastases (stages III–IV) were excluded from our study. We also excluded the patients if their (1) age at initial diagnosis was lower than 18 or higher than 79 years, (2) initial cancer or SPMs were diagnosed *via* death certificate or by postmortem due to unknown survival time, and (3) SPMs occurred within 2 months after initial diagnosis. Next, the qualified patients with early-stage OC were divided into two groups: the SPM cohort and the only one primary malignancy (OOPM) cohort.

### Definition of SPMs

The SEER has detailed and standard rules for the diagnosis of multiple primary tumors, which takes the tumor site of origin, the time duration of diagnosis, laterality of paired organs, tumor behavior (*in situ* vs. invasive), and histological type into consideration (13). Because heightened screening of cancer patients during the initial medical workup tends to identify many simultaneous cancers, a latency of 2 months was set to further discriminate SPMs from initial cancer as proposed by the National Cancer Institute (4).

### Outcome and Variable Declaration

The primary outcome was the occurrence of SPMs after the initial OC diagnosis. Overall survival was defined as the period between the initial cancer diagnosis and death from any cause. SPM overall survival was referred to as the period between the diagnosis of SPMs and death from any cause. The demographic characteristics involved age at initial diagnosis (15–49, 50–64, and 65–79 years), race (white, black, Asian/Pacific Islander, and other), and marital status at initial diagnosis (married/domestic partner, divorced/widowed/separated, and single). Tumor characteristics included laterality (unilateral, bilateral, and contralateral), histology (serous, endometrioid, mucinous, clear cell, Brenner tumor, other epithelial, and non-epithelial) (**Supplementary Table 2**) (14, 15), grade (well differentiated, moderately differentiated, poorly differentiated, and undifferentiated), AJCC 8th stage (stage I and stage II) (12), and the number of lymph nodes examined (<12 and ≥12). Treatment-related variables included surgery (yes and no), chemotherapy (yes and no), and radiotherapy (yes and no).

### Statistical Analysis

The characteristics of the OOPM cohort and the SPM cohort were presented as counts and percentages, and the differences were compared by Pearson’s chi-square test. The Fine and Gray subdistribution hazard regression was used to compute the cumulative incidence of SPMs, and the difference between subgroups was compared by Gray’s test (16). The Fine and Gray model combined with the stepwise elimination method was employed to determine the predictors for SPMs. A competing-risk nomogram based on the predictors screened was constructed to provide an individual prediction on 3-, 5-, and 10-year probabilities of developing SPMs after the initial OC diagnosis. The calibration curves estimated by the bootstrap cross-validation method (500 bootstrap resamples) were plotted to show the accordance of nomogram-predicted probability and observed probability of SPMs. The concordance index (C-index) was also calculated to quantify the predictability of the nomogram. Additionally, decision curve analysis (DCA) was employed to assess the clinical utility of our proposed model (17). The clinical utility was assessed by calculating the net benefit (the weighted sum of true positives minus the sum of false positives) under various screening thresholds. If a predictive model yielded a larger net benefit compared with not applying it during the screening process, it would be considered as clinically useful.

All analyses were conducted by R software (version 4.1.0, R Foundation, Vienna, Austria). The packages “crrstep” and “cmprsk” were used for modeling, and package “rms” was used for nomogram plotting. A two-sided *p*-value < 0.05 was considered statistically significant in all analyses.

## Results

### Characteristics of Patients Enrolled

During a follow-up of up to 20.9 years (median: 8.6 years, interquartile range: 5.5–8.8 years) after initial OC diagnosis, 1,131 (7.9%) patients were diagnosed with subsequent SPMs among 14,314 qualified patients with early-stage OC. SPMs occurred in 60 different sites, and the top 10 sites accounted for 73.1% of the total SPMs. The most frequent sites where SPMs originated were breast, lung and bronchus, thyroid, corpus uteri, and pancreas (**Supplementary Figure 1**).

There was a significant difference between the OOPM cohort and the SPM cohort (**Table 1**). To be specific, the proportion of patients aged 50–64 years at initial diagnosis (44.6% vs. 40.0%, *p* < 0.001) and patients who are white (84.8% vs. 81.3%, *p* = 0.021) in the SPM cohort was higher than that in the OOPM cohort. Patients with a serous histological subtype (27.7% vs. 25.7%, *p* < 0.001) and number of lymph nodes examined <12 (71.2% vs. 67.6%, *p* = 0.015) were more likely to be diagnosed with subsequent SPMs. A higher percentage of patients received surgery (98.9% vs. 97.3%, *p* = 0.003) and radiotherapy (1.9% vs. 1.1%, *p* = 0.023) in the SPM cohort, while the receipt of chemotherapy showed no statistical difference (*p* = 0.472). Additionally, SPMs were more likely to be observed in patients with longer follow-up (39.8% vs. 27.7%, *p* < 0.001). The death rate was higher (43.5% vs. 26.3%, *p* < 0.001), and the main cause of death was subsequent malignancies for patients in the SPM cohort (55.7%), while most patients with OOPM died from the initial OC (73.6%).

**Table 1:** Demographic and clinicopathological characteristics of early-stage ovarian cancer patients with only one primary malignancy or with second primary malignancies.

Variable	Overall, *n* (%)	OOPM cohort, *n* (%)	SPM cohort, *n* (%)	*p*-value
Enrolled	14,314	13,183 (92.10)	1,131 (7.90)	
Age at initial diagnosis, in years				< 0.001
18–49	5,481 (38.29)	5,169 (39.21)	312 (27.59)	
50–64	5,771 (40.32)	5,267 (39.95)	504 (44.56)	
65–79	3,062 (21.39)	2,747 (20.84)	315 (27.85)	
Race				0.021
White	11,638 (81.57)	10,679 (81.29)	959 (84.79)	
Black	1,015 (7.11)	941 (7.16)	74 (6.54)	
Asian/Pacific Islander	1,502 (10.53)	1,411 (10.74)	91 (8.05)	
Other	113 (0.79)	106 (0.81)	7 (0.62)	
Unknown	46	46	0	
Marital status				0.087
Married/domestic partner	7,891 (57.35)	7,278 (57.41)	613 (56.65)	
Divorced/widowed/separated	2,589 (18.82)	2,360 (18.61)	229 (21.16)	
Single	3,280 (23.84)	3,040 (23.98)	240 (22.18)	
Unknown	554	505	49	
Tumor laterality				0.066
Unilateral	12,086 (84.43)	11,133 (81.45)	953 (84.26)	
Bilateral	2,004 (14.00)	1,835 (13.92)	169 (14.94)	
Contralateral	224 (1.56)	215 (1.63)	9 (0.80)	
Histology				<0.001
Serous	3,676 (25.81)	3,364 (25.65)	312 (27.66)	
Endometrioid	3,093 (21.72)	2,797 (21.33)	296 (26.24)	
Mucinous	1,995 (13.73)	1,782 (13.59)	173 (15.34)	
Clear cell	1,799 (12.63)	1,685 (12.85)	114 (10.11)	
Brenner tumor	653 (4.58)	603 (4.60)	50 (4.43)	
Other epithelial*	1,432 (10.05)	1,334 (10.17)	98 (8.69)	
Non-epithelial	1,635 (11.48)	1,550 (11.82)	85 (7.54)	
Unknown	71	68	3	
Tumor grade				0.769
Well differentiated	2,450 (23.70)	2,241 (23.63)	209 (24.53)	
Moderately differentiated	3,228 (31.23)	2,960 (31.21)	268 (31.46)	
Poorly differentiated	3,485 (33.72)	3,198 (33.72)	287 (33.69)	
Undifferentiated	1,173 (11.35)	1,085 (11.44)	88 (10.33)	
Unknown	3978	3699	279	
AJCC 8th stage				0.884
Stage I	10,676 (74.58)	9,835 (74.60)	841 (74.36)	
Stage II	3,638 (25.42)	3,348 (25.40)	290 (25.64)	
Number of lymph nodes examined				0.015
<12	9,335 (67.87)	8,563 (67.58)	772 (71.22)	
≥12	4,420 (32.13)	4,108 (32.42)	312 (28.78)	
Unknown	559	512	47	
Surgery				0.003
No	365 (2.55)	352 (2.67)	13 (1.15)	
Yes	13,940 (97.45)	12,822 (97.33)	1,118 (98.85)	
Unknown	9	9	0	
Chemotherapy				0.472
No	6,871 (48.00)	6,316 (47.91)	555 (49.07)	
Yes	7,443 (52.00)	6,867 (52.09)	576 (50.93)	
Radiotherapy				0.023
No	14,126 (98.87)	13,017 (98.94)	1,109 (98.14)	
Yes	161 (1.13)	140 (1.06)	21 (1.86)	
Unknown	27	26	1	
Year of initial malignancy diagnosis				<0.001
1998–2003	4,102 (28.66)	3,652 (27.70)	450 (39.79)	
2003–2008	5,361 (37.45)	4,942 (37.49)	419 (37.05)	
2008–2013	4,851 (33.89)	4,589 (34.81)	262 (23.17)	
Survival status				<0.001
Alive	10,351 (72.31)	9,712 (73.67)	639 (56.50)	
Dead	3,963 (27.69)	3,471 (26.33)	492 (43.50)	
Cause of death				<0.001
Initial primary malignancy	2,690 (67.88)	2,555 (73.61)	135 (27.44)	
Multiple malignancies	274 (6.91)	0 (0.00)	274 (55.69)	
Non-malignancy cause	934 (23.57)	854 (24.60)	80 (16.26)	
Unknown	65 (1.64)	62 (1.79)	3 (0.61)	

*Other epithelial tumors include carcinosarcoma, large cell, giant cell, spindle cell, pseudo sarcomatous, and mixed histological subtypes.

OOPM, only one primary malignancy; SPMs, second primary malignancies.

### Survival and Cumulative Incidence of SPMs

**Figure 1:**
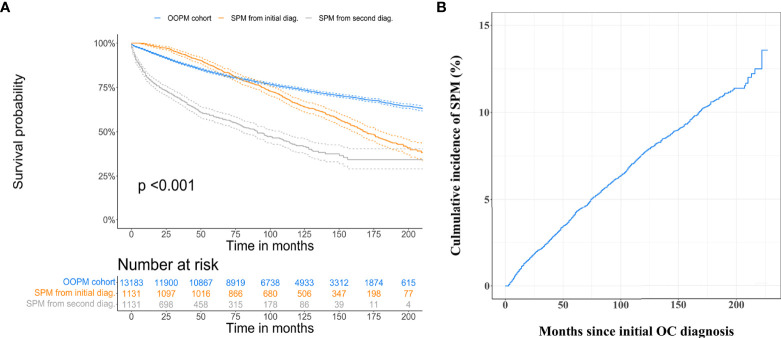
Estimation of overall survival and cumulative incidence of developing second primary malignancies (SPMs). **(A)** Overall survival curves for only one primary malignancy cohort, for the SPM cohort from initial malignancy diagnosis, and second malignancy diagnosis. **(B)** Overall cumulative incidence curve for early-stage ovarian cancer patients developing SPMs after their initial diagnosis, treating non-SPM death as a competing event.

The median overall survival time after initial OC diagnosis for the SPM cohort was 14.0 years, while the OOPM cohort did not reach the median overall survival time. The 5-, 10-, and 15-year overall survival rates for the OOPM cohort versus the SPM cohort were 83.1%, 74.2%, and 66.7% versus 86.2%, 65.5%, and 46.1%, respectively. The SPM cohort had better overall survival than the OOPM cohort within 6.5 years after initial diagnosis; thereafter, the overall survival rates decreased rapidly in the SPM cohort while it continued to steadily decrease in the OOPM cohort. OC patients concurrent with subsequent SPMs experienced a significantly worse prognosis with a median SPM overall survival of 7.6 years, and the 5-, 10-, and 15-year survival rates were merely 58.4%, 42.1%, and 34.3%, respectively (**Figure 1A**). During the follow-up of approximately 19 years, the overall cumulative incidence of SPMs for early-stage OC patients was 13.6%, treating the non-SPM death as a competing event, and the 3-, 5-, and 10-year cumulative incidence were 2.5%, 4.1%, and 7.7%, respectively (**Figure 1B**).

### Predictors for Developing SPMs

**Figure 2:**
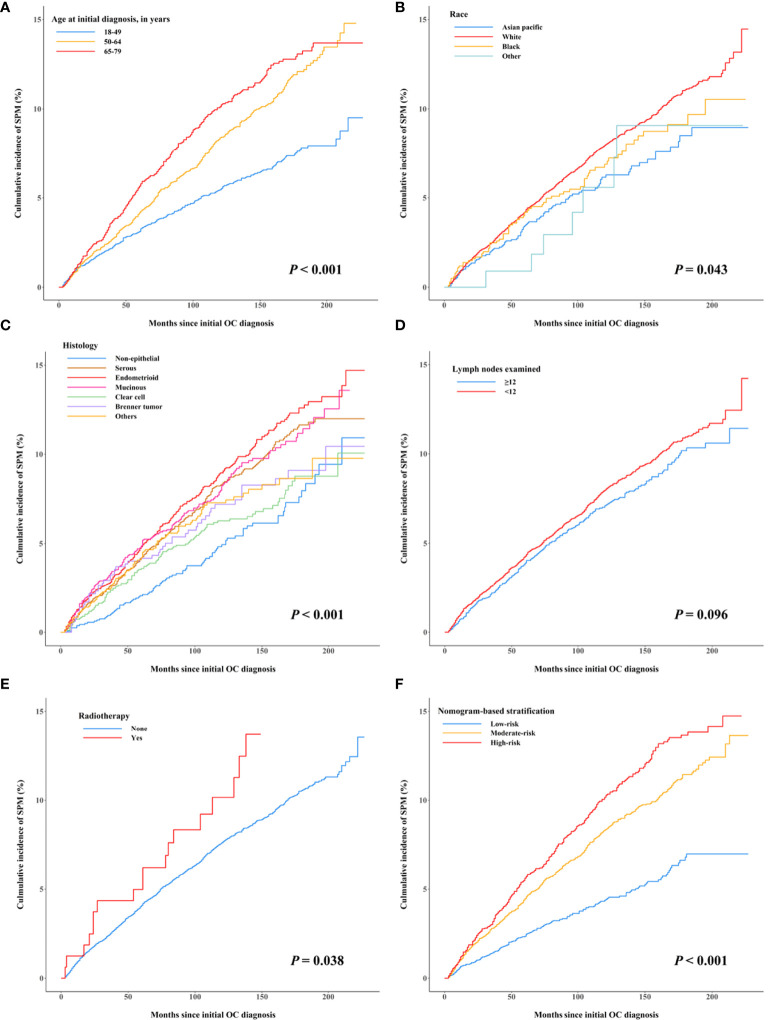
Cumulative incidence curves of early-stage OC patients developing second primary malignancies after their initial diagnosis by subgroups, treating non-SPM death as a competing event. **(A)** Age at initial diagnosis, **(B)** race, **(C)** histology, **(D)** the number of lymph nodes examined, **(E)** radiotherapy, and **(F)** nomogram-based risk stratification.

The multivariable Fine and Gray hazards model combined with the stepwise elimination method was used to evaluate the variables associated with SPM development. After variable selection, five variables, namely, age at initial diagnosis, race, tumor histology, number of lymph nodes examined, and radiotherapy, were retained in the final model (**Table 2** and **Figures 2A–E**). Compared with younger patients (aged 18–49 years), patients aged 50–64 and 65–79 years had substantially increased risks of SPMs, with a subdistribution hazard ratio (sHR) of 1.61 (95% CI = 1.38 to 1.88) and 2.20 (95% CI = 1.85 to 2.62), respectively. White female patients were at an excessive risk of SPMs versus Asian/Pacific Islander female patients (sHR = 1.26, 95% CI = 1.01 to 1.58, *p* = 0.041). Patients with epithelial histologic types, including the serous (sHR = 1.50, 95% CI = 1.16 to 1.95, *p* = 0.002), endometrioid (sHR = 1.58, 95% CI = 1.22 to 2.05, *p* < 0.001), mucinous (sHR = 1.60, 95% CI = 1.22 to 2.09, *p* < 0.001), and Brenner tumor (sHR = 1.46, 95% CI = 1.00 to 2.12, *p* = 0.049) of first primary OC, were related to an elevated risk of developing SPMs. The risk of SPMs was also significantly higher for patients whose number of lymph nodes examined was <12 (sHR = 1.21, 95% CI = 1.06 to 1.39, *p* = 0.004) and who received radiotherapy (sHR = 1.99, 95% CI = 1.29 to 3.05, *p* = 0.002).

**Table 2:** Significant factors associated with second primary malignancies included in the final Fine and Gray subdistribution hazards model.

Variable		sHR	95% CI	*p*-value**
Age at initial diagnosis, in years				
18–49		Reference		
50–64		1.61	1.38–1.88	**<0.001**
65–79		2.20	1.85–2.62	**<0.001**
Race				
Asian/Pacific Islander		Reference		
White		1.26	1.01–1.58	**0.041**
Black		1.35	0.99–1.85	0.059
Other		0.95	0.42–2.17	0.905
Histology				
Non-epithelial	Reference			
Serous	1.50		1.16–1.95	**0.002**
Endometrioid	1.58		1.22–2.05	**<0.001**
Mucinous	1.60		1.22–2.09	**<0.001**
Clear cell	1.14		0.85–1.54	0.380
Brenner tumor	1.46		1.00–2.12	**0.049**
Other epithelial*	1.35		1.00–1.84	0.052
Number of lymph nodes examined				
≥12	Reference			
<12	1.21		1.06–1.39	**0.004**
Radiotherapy				
No	Reference			
Yes	1.99		1.29–3.05	**0.002**

*Other epithelial tumors include carcinosarcoma, large cell, giant cell, spindle cell, pseudo sarcomatous, and mixed histological subtypes. **The bold values of p-value indicate statistically significant.

sHR, subdistribution hazard ratio; 95% CI, 95% confidence interval.

Potential covariates included age at initial diagnosis, race, marital status, tumor laterality, histology, grade, AJCC 8th stage, number of lymph nodes examined, surgery, chemotherapy, and radiotherapy.

All variables with unknown data were removed in multivariable competing risk regression analyses. Bold values, It implies statistically significant (P<0.05).

### Competing-Risk Nomogram Construction, Risk Stratification, and Evaluation

**Figure 3:**
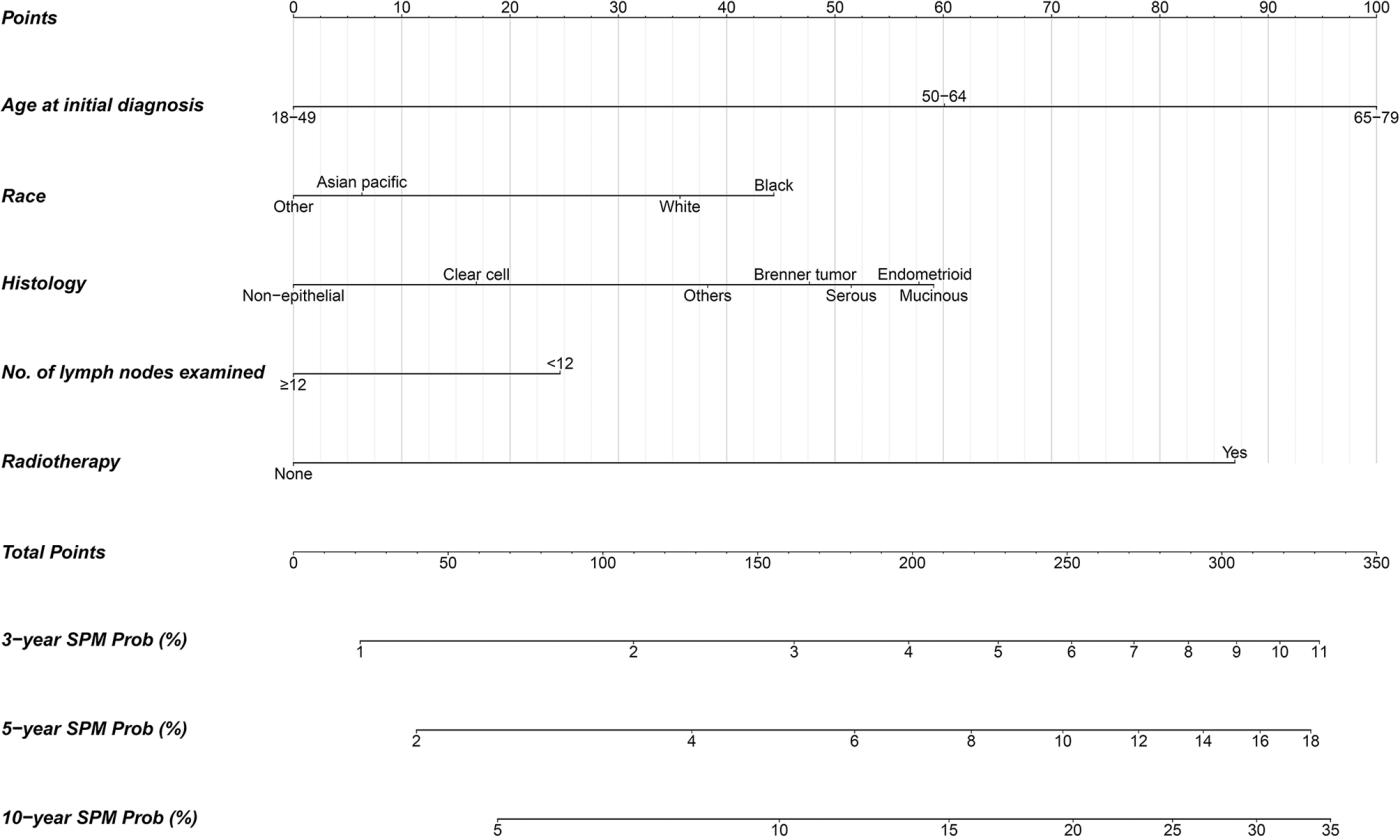
Competing-risk nomogram for predicting 3-, 5-, and 10-year probabilities of developing second primary malignancies in early-stage OC patients.

**Figure 4:**
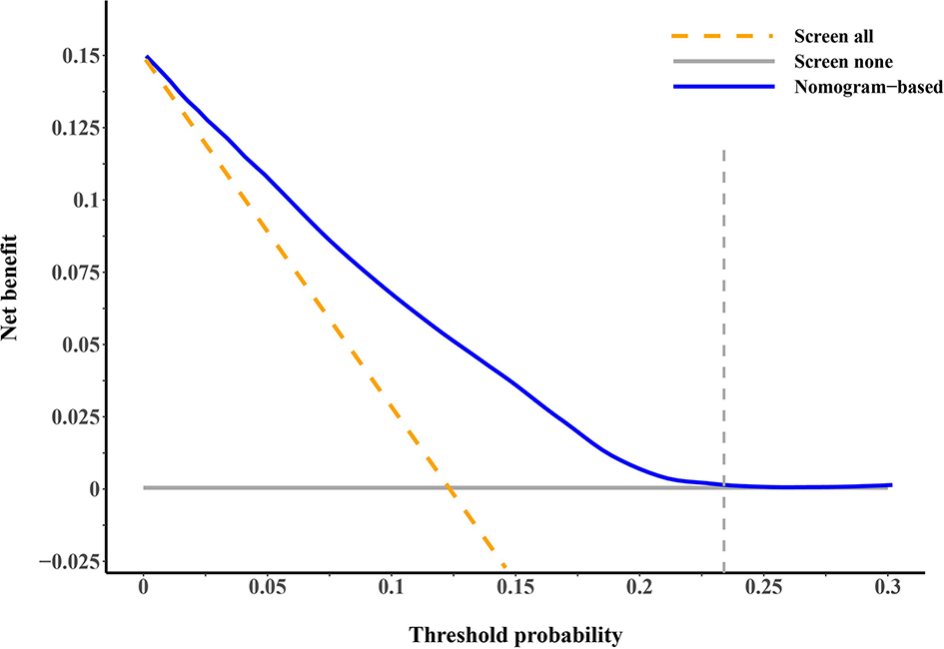
Decision curve analysis for evaluating the clinical utility of our proposed competing-risk nomogram. The *x*-axis is the threshold probability for screening of second primary malignancies (SPMs) and the *y*-axis is the corresponding net benefit. The blue solid line depicts the net benefits change of screening using our proposed nomogram under different threshold probabilities, whereas the gold dashed line and gray solid line represent the net benefit of screening all and screening no patients. As the decision curve noted, using the nomogram as the screening tool when the threshold probability was between 1% and 23% would obtain more clinical benefits than simply screening all patients or screening no one.

A nomogram integrating the above-mentioned risk predictors was established to calculate the total risk points and corresponding SPM probabilities of 3-, 5-, and 10-years (**Figure 3**). The patients were grouped into low risk, intermediate risk, and high risk of SPMs according to the total points calculated by the nomogram. The detailed point of each value for the risk predictors and of each nomogram-based risk group is listed in **Supplementary Table 3**. The cumulative incidence of SPMs was statistically different among the nomogram-based low-risk, intermediate-risk, and high-risk groups, especially for low-risk versus high-risk groups (**Figure 2F**). Evaluation of the nomogram showed a moderate discriminatory power with a bootstrap-corrected C-index of 68.1% (95% CI = 67.9 to 68.3%). Calibration results also indicated good consistency between the nomogram predicted probability and the observed probability of 3-year, 5-year, and 10-year SPMs, as curves got close to the 45° diagonal line (**Supplementary Figures 2**–**4**). Additionally, the result of DCA demonstrated that when the screening threshold of SPMs was given between 1% and 23%, the clinical net benefit would be higher using the proposed nomogram as the screening tool compared to the strategies of screening all patients or screening no one (**Figure 4**).

## Discussion

In this population-based cohort study, we found that the cumulative incidence of SPMs among primary early-stage OC survivors was 13.6% at the maximum follow-up of 20.9 years. Age at initial cancer diagnosis, race, histologic subtype of OC, number of lymph nodes examined, and radiotherapy were shown to be the risk factors of developing SPMs. For clinical convenience, a competing-risk nomogram was proposed to quantitatively predict the probabilities of developing SPMs in succeeding years, and its clinical utility was confirmed by DCA.

During the entire follow-up period, approximately 83 in 1,000 patients were observed to develop a subsequent malignancy, which is much higher than the standardized incidence of malignancy in US women of 4 in 1,000 (18).

The patients with SPMs tended to be older and white, and to have received surgery and radiotherapy. Initially, the prognosis for the SPM cohort was better than the OOPM cohort; however, an obvious survival disadvantage was observed during the follow-up. Some possible reasons were as follows: (1) some patients developed new malignancies that were more advanced and more lethal than the initial early-stage malignancy (19); (2) in addition to the presence of higher tumor burden, patients with SPMs also experienced higher psychological distress (20), which has a negative impact on their health and compliance with medical advice; and (3) patients in the SPM cohort tended to be older, so the treatment options were more likely to be conservative compared to younger patients (21). The breast was the most common site of a new malignancy for early-stage OC; this phenomenon might be due to (1) breast cancer being the most common cancer in women, and (2) the fact that the occurrence of ovarian and breast cancers shares the same risk exposure of a genetic mutation in either BRCA1 or BRCA2 (22, 23). Additionally, evidence supported the importance of endogenous hormones in the etiology of ovarian and breast cancers; the high incidence of breast cancer after OC might also be attributed to some shared hormone exposures (24). Our study suggested that the lung and bronchus, thyroid, and corpus uteri were also common sites for developing SPMs, although the underlying mechanisms remain to be examined. This finding may suggest risk-specific screening strategies at follow-up for sites with different risks of SPM development (25).

Quantifying the association between demographic as well as clinicopathological factors and SPM development to identify high-risk individuals are of both clinical and public health significance. In consideration of a considerable proportion of early-stage OC patients who died before developing SPMs, we employed the competing risk model to unbiasedly estimate the effect of factors of interest. The multivariable analysis revealed that older age at initial diagnosis, white race, epithelial histologic subtype (serous, endometrioid, mucinous, and Brenner tumor), number of lymph nodes examined <12, and radiotherapy were significantly associated with an elevated risk of SPMs. Older age and white race have been reported to be risk factors for SPMs in OC (6) or other cancers (26, 27). From an epidemiologic perspective, the incidence of cancer is higher in the older population due to the weakening of immune monitoring, reduced gene repair, variations in estrogen secretion and its receptor sensitivity, and reduced tolerance to carcinogens (28, 29). The etiology of the effect difference on race might be multifactorial and might encompass a diverse genetic background, various treatment options for OC, coverage of healthcare, different economic conditions, and living habits. The serous OC was shown to be associated with an increased risk of subsequent primary leukemia previously (30). Schrader et al. (31) found that more frequent BRCA1 and BRCA2 mutations were presented in the serous histology of OC, and patients with these mutations were prone to develop SPMs (22, 23). Previous studies also found a relationship between increased risk of SPMs and endometrioid OC (5, 32), as well as between increased risk of SPMs and mucinous OC (5). Their findings supported our results although the pathological mechanism has not been well studied. A study was performed to recommend the least number of lymph nodes examined for node staging of gastric cancer (33). A study reported that a higher number of lymph nodes examined was associated with a higher rate of nodal metastasis detected in colorectal cancer (34). Some patients with stages III or IV OC were possibly misdiagnosed as stages I or II in our dataset due to an insufficient number of lymph nodes examined. A case–control study showed that patients with advanced-stage (stages III and IV) head and neck cancer have a significantly higher risk of SPM of the esophagus compared to those with early stage (stages I and II) (35). This evidence provides a potential explanation of the association between the number of lymph nodes examined <12 and a higher risk of SPMs in early-stage OC. However, due to the heterogeneity between cancers, further studies are needed to provide direct evidence to validate our hypothesis. Radiotherapy uses ionization to kill tumor tissues, but also causes genetic mutations in normal cells (36), and the long-term negative effect of radiotherapy, including secondary tumorigenesis, has been broadly reported previously (37–39). Radiotherapy is rarely used to treat early-stage OC. Benefiting from a national-based cancer database analysis, our results showed that radiotherapy is associated with a higher risk of SPMs. This finding adds to the evidence that patients with early-stage OC should be cautious about radiotherapy. Thus, physicians ought to cautiously weigh the clinical benefits against the latent threats when scheduling radiotherapy to early-stage OC patients. Additionally, we also observed an association between the histological subtype of Brenner tumor of OC and an increased risk of SPMs, which had not been observed previously. However, further studies are needed to confirm this association.

Current surveillance procedures for OC mainly focused on its early detection (40), recurrence (41), and metastases (42), with little attention paid to the screening of subsequent malignancies. However, the risk of SPM development could not be neglected for patients with early-stage OC according to our findings. Therefore, we established a practical scoring tool named the competing-risk nomogram to estimate the risk of SPMs for early-stage OC patients in the existence of the competing event of non-SPM death. The nomogram provided individual total risk points and the prediction of 3-, 5-, and 10-year SPM probabilities if the required indicators were given. The patients could be further stratified into different risk levels according to their total risk points, and different screening strategies should be considered for patients with different risk levels during follow-up. A previous study has shown that OC patients treated in high-volume hospitals have a lower mortality rate, which may be attributed to the high quality of treatment and care provided by gynecologic oncologists (43). Patients with early-stage OC who have a high risk of SPMs would have a better chance of receiving high-quality treatment and care if they were treated at high-volume hospitals. This may be able to improve their survival. Given that breast, lung and bronchus, and thyroid cancers account for a high proportion of all SPMs, more attention could be paid to these sites during screening to detect the lesions as early as possible. DCA demonstrated the clinical utility of our proposed nomogram as it produced a superior net benefit compared to that in hypothetical all-screening or no-screening conditions.

Our study has several strengths. The data from a large population-based cohort collected by multiple registries effectively avoid selection and referral biases. All SEER registries comply with the strict criteria of data collection and follow-up, which ensures the quality of our data. We included the largest amount of early-stage OC patients with SPMs to date, and unbiasedly estimated the risk factors of SPMs for the first time. Our proposed nomogram has relatively good discrimination to identify early-stage OC patients with different SPM risks.

There are also some limitations. First, some acknowledged factors related to the formation of tumors, such as smoking status, comorbidities, family history of cancer, diet habit, and lifestyles, are not available in the SEER database. Second, the difference in chemotherapy regimens that are possibly related to SPM occurrence, such as type of chemotherapy, use of neoadjuvant chemotherapy, use of adjuvant chemotherapy, and maintenance chemotherapy or length thereof after primary chemotherapy, may improve the performance of the prediction model but such information is not available in the SEER database. Third, surgical rupture of the ovarian capsule has been reported to be a factor limiting survival in patients with early-stage OC (44), and this factor may also influence the development of SPMs. However, the information on surgical complications is not available in the SEER database and further research is warranted. The moderate C-index observed in our proposed nomogram might partly be attributed to the lack of these data. Fourth, some of the patients who did not receive surgical treatment may have been misclassified as early-stage OC as their TNM stage is mostly determined by clinical evidence without pathological evaluation. However, because the vast majority of the patients (97.45%) in our study received surgery, their stage of OC would not affect our findings. Some cases of recurrent or metastatic OC might have been misclassified as SPMs. Nevertheless, the SEER program has a detailed definition of SPMs and strict procedures for the diagnosis of SPMs (13). A 2-month interval exclusion was used to further discriminate the SPMs from the initial simultaneous malignancies (4). Moreover, although our proposed nomogram has been validated by the bootstrap method, it still needs to be further verified with external cohorts from other sources. More potential factors could be considered to establish a predictive model with better performance. Finally, a comprehensive analysis of various most vulnerable SPM sites for early-stage OC is needed to help develop more targeted follow-up strategies.

## Conclusion

In conclusion, early-stage OC survivors remain at a high risk of developing SPMs. Older age at initial diagnosis, white race, number of lymph nodes examined (<12), radiotherapy, and histological diagnosis of serous, endometrioid, mucinous, and Brenner tumors were related to an elevated risk of SPMs for early-stage OC. A user-friendly competing-risk nomogram for predicting the 3-, 5-, and 10-year SPM probabilities was constructed, which could be useful in helping clinicians evaluate the SPM risk for early-stage OC patients and arrange their future screening program for SPMs. Since our study was not validated by external data, further studies to establish the formal documents of surveillance and screening for SPMs are still needed.

## Data Availability Statement

Publicly available datasets were analyzed in this study. These data can be found here: https://seer.cancer.gov/data-software/.

## Ethics Statement

Ethical review and approval were not required for the study on human participants in accordance with the local legislation and institutional requirements. Written informed consent for participation was not required for this study in accordance with the national legislation and the institutional requirements.

## Author Contributions

YY, XZ, and GQ conceptualized and designed the study. JX, CH, and ZW collected the data and performed the statistical analysis. JX wrote the manuscript. HX, JL, YC, CW, and JZ reviewed the literature and revised the manuscript. All authors contributed to the article and approved the submitted version.

## Funding

This work was funded by the Natural Science Foundation of China (82173612 and 82173613), the Shanghai Rising-Star Program (21QA1401300), the Shanghai Municipal Natural Science Foundation (22ZR1414900), and the Three-year Action Program of Shanghai Municipality for Strengthening the Construction of Public Health System (GWV-10.1-XK05) Big Data and Artificial Intelligence Application and Project supported by Shanghai Municipal Science and Technology Major Project (ZD2021CY001).

## Conflict of Interest

The authors declare that the research was conducted in the absence of any commercial or financial relationships that could be construed as a potential conflict of interest.

## Publisher’s Note

All claims expressed in this article are solely those of the authors and do not necessarily represent those of their affiliated organizations, or those of the publisher, the editors and the reviewers. Any product that may be evaluated in this article, or claim that may be made by its manufacturer, is not guaranteed or endorsed by the publisher.

## Supplementary Material

Supplementary Figure 1 Top 10 most frequent sites of developing second primary malignancies in early-stage ovarian cancer patients.

Supplementary Figure 2 Calibration curves for 3-year predicted vs. observed probability of developing second primary malignancies estimated by the Fine and Gray model.

Supplementary Figure 3 Calibration curves for 5-year predicted vs. observed probability of developing second primary malignancies estimated by the Fine and Gray model.

Supplementary Figure 4 Calibration curves for 10-year predicted vs. observed probability of developing second primary malignancies estimated by the Fine and Gray model.

## Abbreviations

OC, ovarian cancer; SPMs, second primary malignancies; SEER, Surveillance, Epidemiology and End Results; DCA, decision curve analysis; OOPM, only one primary malignancy; CI, confidence interval; sHR, subdistribution hazard ratio.

## References

[1] Siegel RL Miller KD Fuchs HE Jemal A. Cancer Statistics, 2022. CA: Cancer J Clin (2022) 72(1):7–33. doi: 10.3322/caac.21708 35020204

[2] Torre LA Trabert B DeSantis CE Miller KD Samimi G Runowicz CD. Ovarian Cancer Statistics, 2018. CA: Cancer J Clin (2018) 68(4):284–96. doi: 10.3322/caac.21456 PMC6621554 29809280

[3] Donin N Filson C Drakaki A Tan HJ Castillo A Kwan L. Risk of Second Primary Malignancies Among Cancer Survivors in the United States, 1992 Through 2008. Cancer (2016) 122(19):3075–86. doi: 10.1002/cncr.30164 PMC6192520 27377470

[4] Curtis RE Freedman DM Ron E Ries LAG Hacker DG Edwards BK. New Malignancies Among Cancer Survivors: Seer Cancer Registries, 1973-2000. Bethesda, Maryland: National Cancer Institute, NIH Publ. No.05-5302 (2006).

[5] Kanninen TT Nasioudis D Sisti G Holcomb K Di Tommaso M Khalil S. Epidemiology of Second Primary Tumors in Women With Ovarian Cancer. Int J Gynecol Cancer (2017) 27(4):659–67. doi: 10.1097/IGC.0000000000000950 28441249

[6] Hung Y-P Liu C-J Hu Y-W Chen M-H Li C-P Yeh C-M. Secondary Primary Malignancy Risk in Patients With Ovarian Cancer in Taiwan: A Nationwide Population-Based Study. Medicine (2015) 94(38):e1626. doi: 10.1097/MD.0000000000001626 PMC4635773 26402833

[7] Dent SF Klaassen D Pater JL Zee B Whitehead M. Second Primary Malignancies Following the Treatment of Early Stage Ovarian Cancer: Update of a Study by the National Cancer Institute of Canada—Clinical Trials Group (Ncic—Ctg). Ann Oncol (2000) 11(1):65–8. doi: 10.1023/A:1008356806417 10690389

[8] Kaldor JM Day NE Pettersson F Clarke EA Pedersen D Mehnert W. Leukemia Following Chemotherapy for Ovarian Cancer. New Engl J Med (1990) 322(1):1–6. doi: 10.1056/NEJM199001043220101 2104664

[9] Zheng G Chattopadhyay S Försti A Sundquist K Hemminki K. Familial Risks of Second Primary Cancers and Mortality in Ovarian Cancer Patients. Clin Epidemiol (2018) 10:1457. doi: 10.2147/CLEP.S174173 30349393 PMC6188204

[10] Kim HT. Cumulative Incidence in Competing Risks Data and Competing Risks Regression Analysis. Clin Cancer Res (2007) 13(2):559–65. doi: 10.1158/1078-0432.CCR-06-1210 17255278

[11] Surveillance, Epidemiology, and End Results (Seer) Program Research Data (1975–2018). National CancerInstitute, DCCPS. Available at: http://www.seer.cancer.gov/ (Accessed 2021 30 October).

[12] Amin MB Edge S Greene F Byrd DR Brookland RK Washington MK. AJCC Cancer Staging Manual. 8 ed. New York: Springer International Publishing (2017).

[13] Johnson C Peace S Adamo P Fritz A Percy-Laurry A Edwards BK. Multiple Primary and Histology Coding Rules. Bethesda, Maryland: National Cancer Institute Surveillance Epidemiology and End Results Program (2007).

[14] Kurman RJ Carcangiu ML Herrington CS Young RH. Who Classification of Tumours of Female Reproductive Organs. Lyon: International Agency for Research on Cancer (2014).

[15] Peres LC Cushing-Haugen KL Kobel M Harris HR Berchuck A Rossing MA. Invasive Epithelial Ovarian Cancer Survival by Histotype and Disease Stage. J Natl Cancer Inst (2019) 111(1):60–8. doi: 10.1093/jnci/djy071 PMC6335112 29718305

[16] Fine JP Gray RJ. A Proportional Hazards Model for the Subdistribution of a Competing Risk. J Am Stat Assoc (1999) 94(446):496–509. doi: 10.1080/01621459.1999.10474144

[17] Vickers AJ Elkin EB. Decision Curve Analysis: A Novel Method for Evaluating Prediction Models. Med Decision Making (2006) 26(6):565–74. doi: 10.1177/0272989X06295361 PMC2577036 17099194

[18] Henley SJ Ward EM Scott S Ma J Anderson RN Firth AU. Annual Report to the Nation on the Status of Cancer, Part I: National Cancer Statistics. Cancer (2020) 126(10):2225–49. doi: 10.1002/cncr.32802 PMC7299151 32162336

[19] Holland JM Arsanjani A Liem BJ Hoffelt SC Cohen JI Stevens KR. Second Malignancies in Early Stage Laryngeal Carcinoma Patients Treated With Radiotherapy. J Laryngol Otol (2002) 116(3):190–3. doi: 10.1258/0022215021910500 11893260

[20] Belcher SM Hausmann EA Cohen SM Donovan HS Schlenk EA. Examining the Relationship Between Multiple Primary Cancers and Psychological Distress: A Review of Current Literature. Psycho-Oncology (2017) 26(12):2030–9. doi: 10.1002/pon.4299 27758055

[21] Schuurman MS Kruitwagen R Portielje JEA Roes EM Lemmens V van der Aa MA. Treatment and Outcome of Elderly Patients With Advanced Stage Ovarian Cancer: A Nationwide Analysis. Gynecol Oncol (2018) 149(2):270–4. doi: 10.1016/j.ygyno.2018.02.017 29514738

[22] Golmard L Delnatte C Laugé A Moncoutier V Lefol C Abidallah K. Breast and Ovarian Cancer Predisposition Due to *De Novo* Brca1 and Brca2 Mutations. Oncogene (2016) 35(10):1324–7. doi: 10.1038/onc.2015.181 26028024

[23] Metcalfe KA Lynch HT Ghadirian P Tung N Olivotto IA Foulkes WD. The Risk of Ovarian Cancer After Breast Cancer in Brca1 and Brca2 Carriers. Gynecol Oncol (2005) 96(1):222–6. doi: 10.1016/j.ygyno.2004.09.039 15589605

[24] Eliassen AH Hankinson SE. Endogenous Hormone Levels and Risk of Breast, Endometrial and Ovarian Cancers: Prospective Studies. Adv Exp Med Biol (2008) 630:148–65. doi: 10.1007/978-0-387-78818-0_10 18637490

[25] Haughey BH Arfken CL Gates GA Harvey J. Meta-Analysis of Second Malignant Tumors in Head and Neck Cancer: The Case for an Endoscopic Screening Protocol. Ann Otol Rhinol Laryngol (1992) 101(2):105–12. doi: 10.1177/000348949210100201 1531402

[26] Jia H Li Q Yuan J Sun X Wu Z. Second Primary Malignancies in Patients With Colorectal Cancer: A Population-Based Analysis. Oncologist (2020) 25(4):e644. doi: 10.1634/theoncologist.2019-0266 PMC7160402 31943509

[27] Chao C Bhatia S Xu L Cannavale KL Wong FL Huang P-YS. Incidence, Risk Factors, and Mortality Associated With Second Malignant Neoplasms Among Survivors of Adolescent and Young Adult Cancer. JAMA Netw Open (2019) 2(6):e195536–e. doi: 10.1001/jamanetworkopen.2019.5536 PMC6563559 31173129

[28] Miller RA. Aging and Immune Function. Int Rev Cytol (1991) 124:187–215. doi: 10.1016/S0074-7696(08)61527-2 2001916

[29] Chen GG Zeng Q Tse GMK. Estrogen and Its Receptors in Cancer. Medicinal Res Rev (2008) 28(6):954–74. doi: 10.1002/med.20131 18642351

[30] Lim MC Won Y-J Lim J Salehi T Yoo CW Bristow RE. Second Primary Cancer After Primary Peritoneal, Epithelial Ovarian, and Fallopian Tubal Cancer: A Retrospective Study. BMC Cancer (2018) 18(1):1–8. doi: 10.1186/s12885-018-4700-3 30089478 PMC6083613

[31] Schrader KA Hurlburt J Kalloger SE Hansford S Young S Huntsman DG. Germline Brca1 and Brca2 Mutations in Ovarian Cancer: Utility of a Histology-Based Referral Strategy. Obstet Gynecol (2012) 120(2 Part 1):235–40. doi: 10.1097/AOG.0b013e31825f3576 22776961

[32] van Niekerk CC van Dijck JAAM Verbeek ALM. The Impact of Histological Subtype in Developing Both Ovarian and Endometrial Cancer: A Longstanding Nationwide Incidence Study. Eur J Obstet Gynecol Reprod Biol (2018) 221:17–22. doi: 10.1016/j.ejogrb.2017.12.014 29227847

[33] Lee HK Yang HK Kim WH Lee KU Choe KJ Kim JP. Influence of the Number of Lymph Nodes Examined on Staging of Gastric Cancer. J Br Surg (2001) 88(10):1408–12. doi: 10.1046/j.0007-1323.2001.01875.x 11578301

[34] Kim J Huynh R Abraham I Kim E Kumar RR. Number of Lymph Nodes Examined and Its Impact on Colorectal Cancer Staging. Am Surgeon (2006) 72(10):902–5. doi: 10.1177/000313480607201013 17058731

[35] Chung C-S Liao L-J Lo W-C Chou Y-H Chang Y-C Lin Y-C. Risk Factors for Second Primary Neoplasia of Esophagus in Newly Diagnosed Head and Neck Cancer Patients: A Case–Control Study. BMC Gastroenterol (2013) 13(1):1–9. doi: 10.1186/1471-230X-13-154 24456340 PMC4028981

[36] De Ruysscher D Niedermann G Burnet NG Siva S Lee AWM Hegi-Johnson F. Radiotherapy Toxicity. Nat Rev Dis Primers (2019) 5(1):1–20. doi: 10.1038/s41572-019-0064-5 30792503

[37] Marcheselli R Marcheselli L Cortesi L Bari A Cirilli C Pozzi S. Risk of Second Primary Malignancy in Breast Cancer Survivors: A Nested Population-Based Case-Control Study. J Breast Cancer (2015) 18(4):378–85. doi: 10.4048/jbc.2015.18.4.378 PMC4705090 26770245

[38] Yang Y Yang Y Yan S. Risk and Survival of Second Primary Malignancies Following Diagnosis of Gastric Mucosa-Associated Lymphoid Tissue Lymphomas: A Population-Based Study. Curr Problems Cancer (2021) 45(6):100735. doi: 10.1016/j.currproblcancer.2021.100735Ge 33867153

[39] Arnold M Liu L Kenter GG Creutzberg CL Coebergh JW Soerjomataram I. Second Primary Cancers in Survivors of Cervical Cancer in the Netherlands: Implications for Prevention and Surveillance. Radiother Oncol (2014) 111(3):374–81. doi: 10.1016/j.radonc.2014.04.011 24833558

[40] Nebgen DR Lu KH Bast RC. Novel Approaches to Ovarian Cancer Screening. Curr Oncol Rep (2019) 21(8):1–11. doi: 10.1007/s11912-019-0816-0 PMC6662655 31346778

[41] Chen Y-m Chen T Zee C-S Shi Y-p Wan L-r Tong L-j. Is There an Impact of 18f-Fdg Pet/Ct on the Surveillance and Clinical Management of Recurrent Ovarian Cancer? Research Based on a Large Sample in a Single Pet/Ct Center. Nucl Med Commun (2014) 35(4):347–52. doi: 10.1097/MNM.0000000000000051 24257481

[42] Ai Y Zhang J Jin J Zhang J Zhu H Jin X. Preoperative Prediction of Metastasis for Ovarian Cancer Based on Computed Tomography Radiomics Features and Clinical Factors. Front Oncol (2021) 11:610742. doi: 10.3389/fonc.2021.610742 34178617 PMC8222738

[43] Wright JD Herzog TJ Siddiq Z Arend R Neugut AI Burke WM. Failure to Rescue as a Source of Variation in Hospital Mortality for Ovarian Cancer. J Clin Oncol (2012) 30(32):3976–82. doi: 10.1200/JCO.2012.43.2906 23032619

[44] Matsuo K Huang Y Matsuzaki S Klar M Roman LD Sood AK. Minimally Invasive Surgery and Risk of Capsule Rupture for Women With Early-Stage Ovarian Cancer. JAMA Oncol (2020) 6(7):1110–3. doi: 10.1001/jamaoncol.2020.1702 PMC7290694 32525512

