# Supplementary figures and images for: Risk Prediction of Second Primary Malignancies in Primary Early-Stage Ovarian Cancer Survivors: A SEER-Based National Population-Based Cohort Study
Source: Front Oncol. 2022 May 19;12:875489. doi: 10.3389/fonc.2022.875489 (PMC9161780; doi:10.3389/fonc.2022.875489)

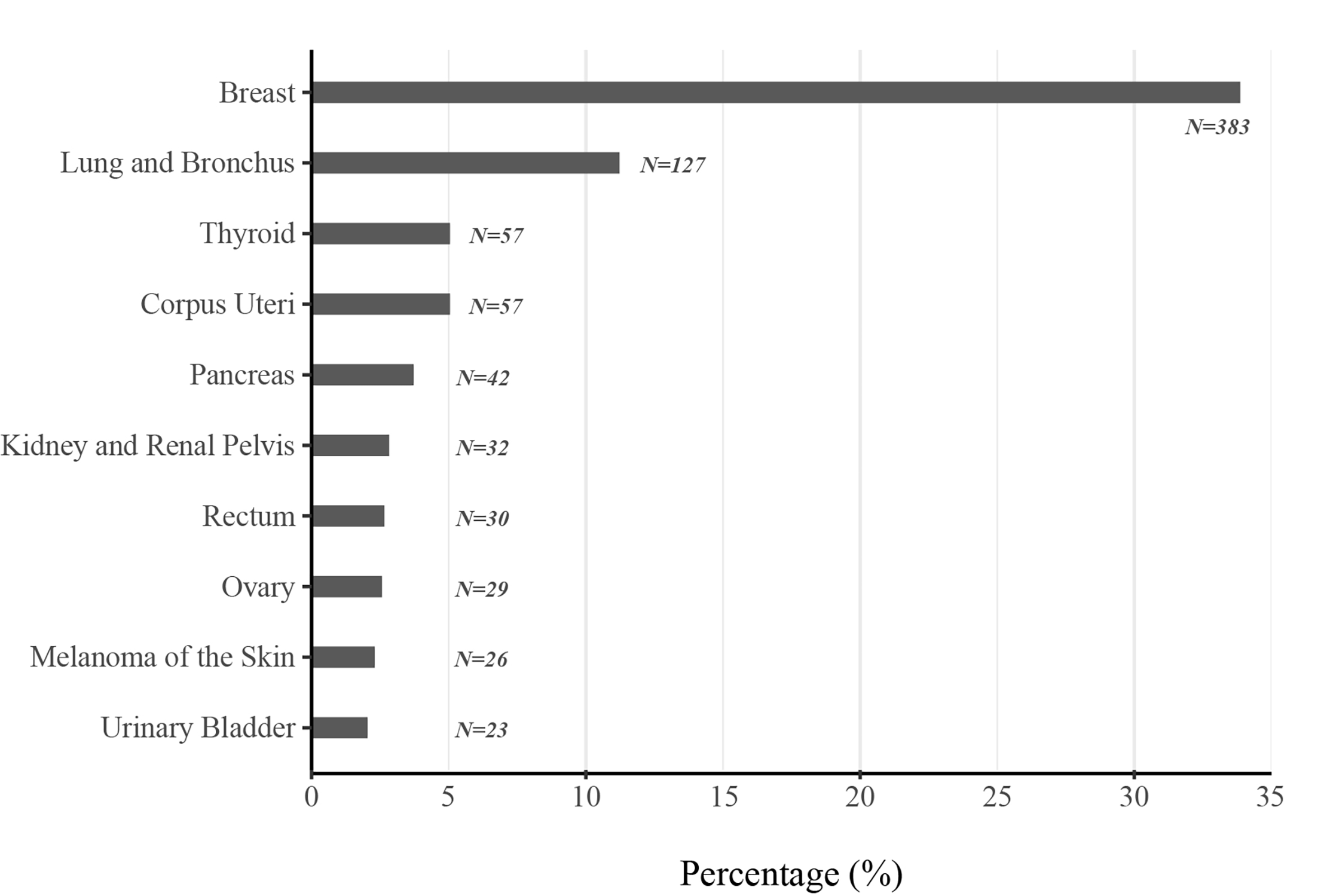

Supplement: Supplementary Figure 1 — Top 10 most frequent sites of developing second primary malignancies in early-stage ovarian cancer patients. [file Image_1.tif]

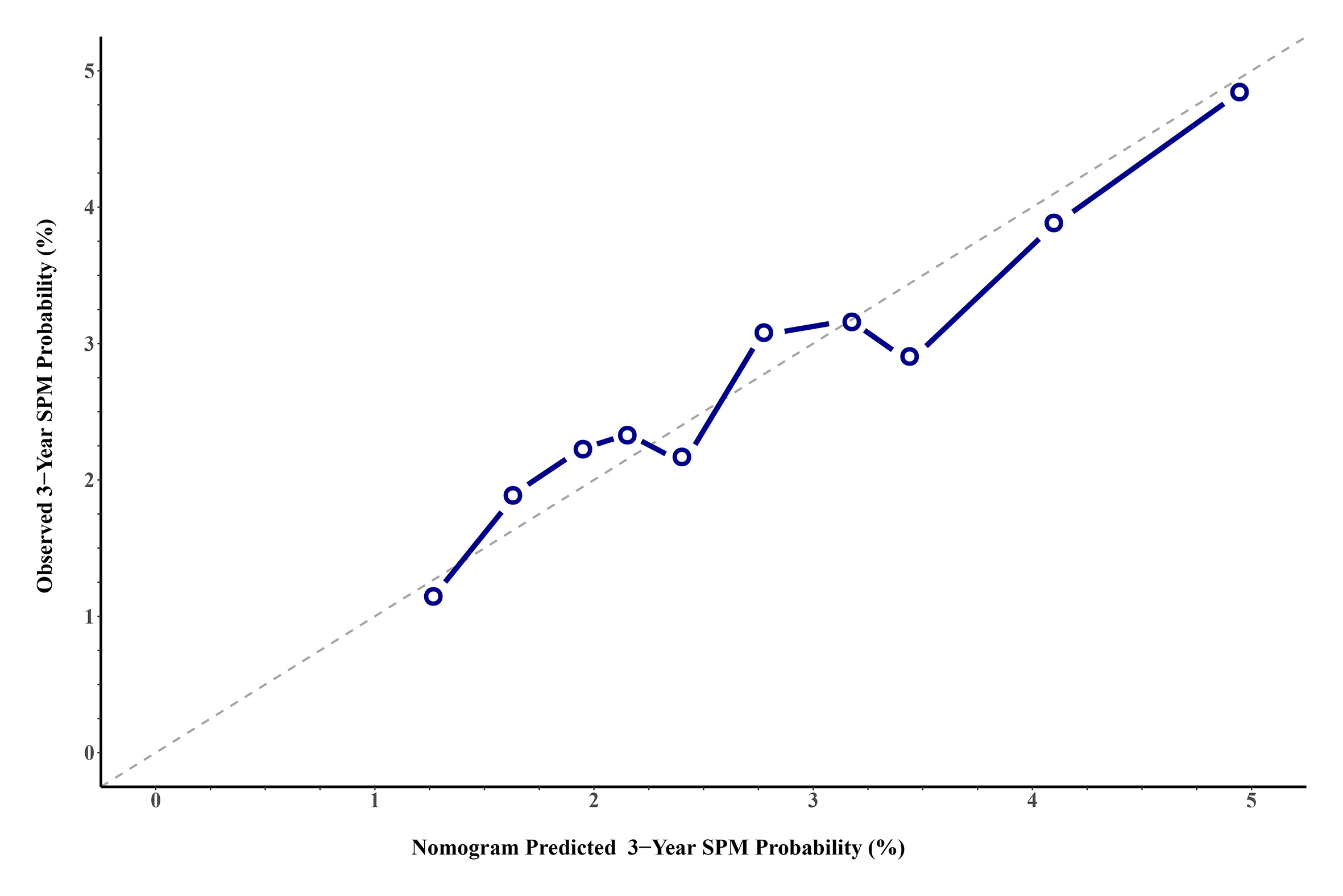

Supplement: Supplementary Figure 2 — Calibration curves for 3-year predicted vs. observed probability of developing second primary malignancies estimated by the Fine and Gray model. [file Image_2.tif]

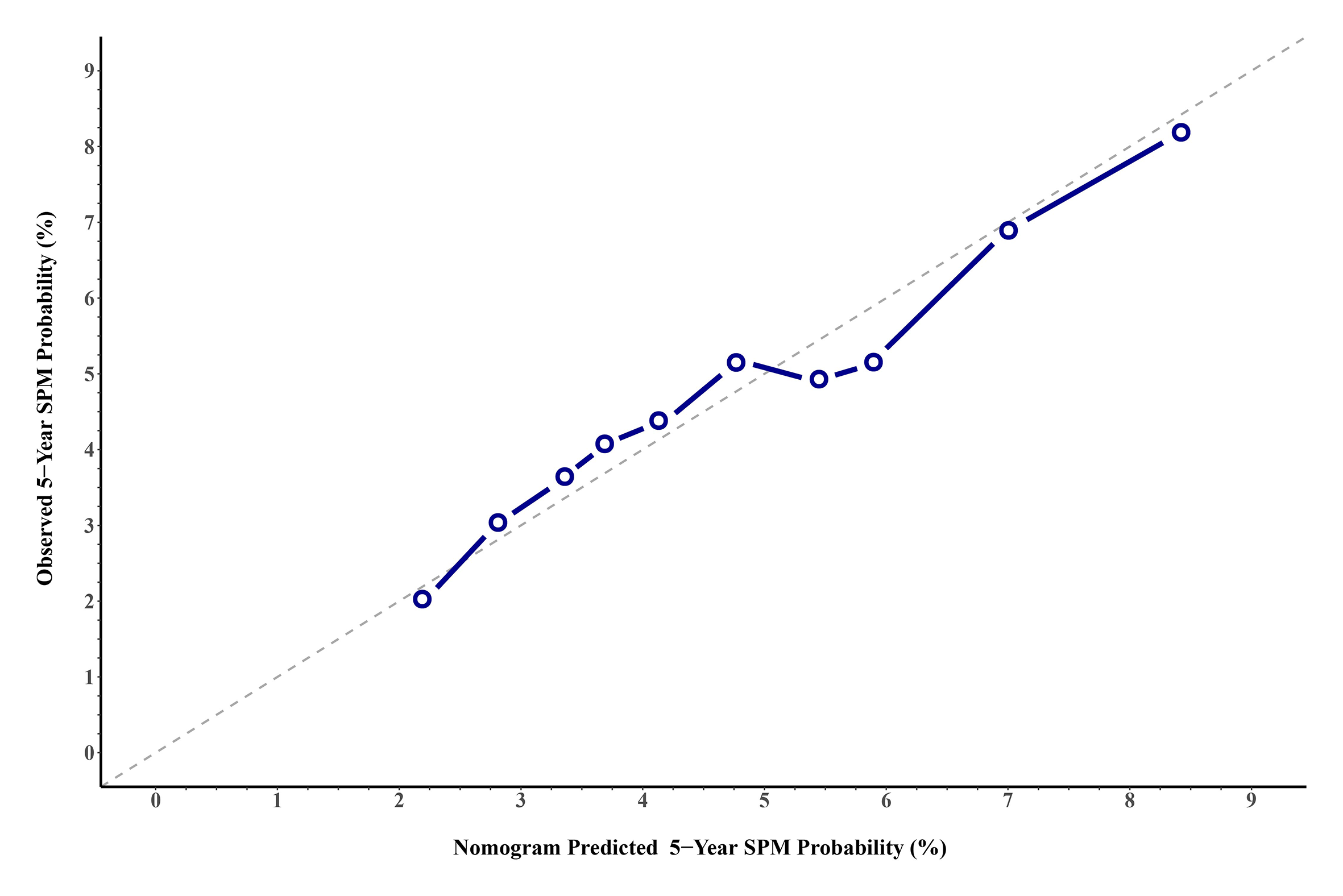

Supplement: Supplementary Figure 3 — Calibration curves for 5-year predicted vs. observed probability of developing second primary malignancies estimated by the Fine and Gray model. [file Image_3.tif]

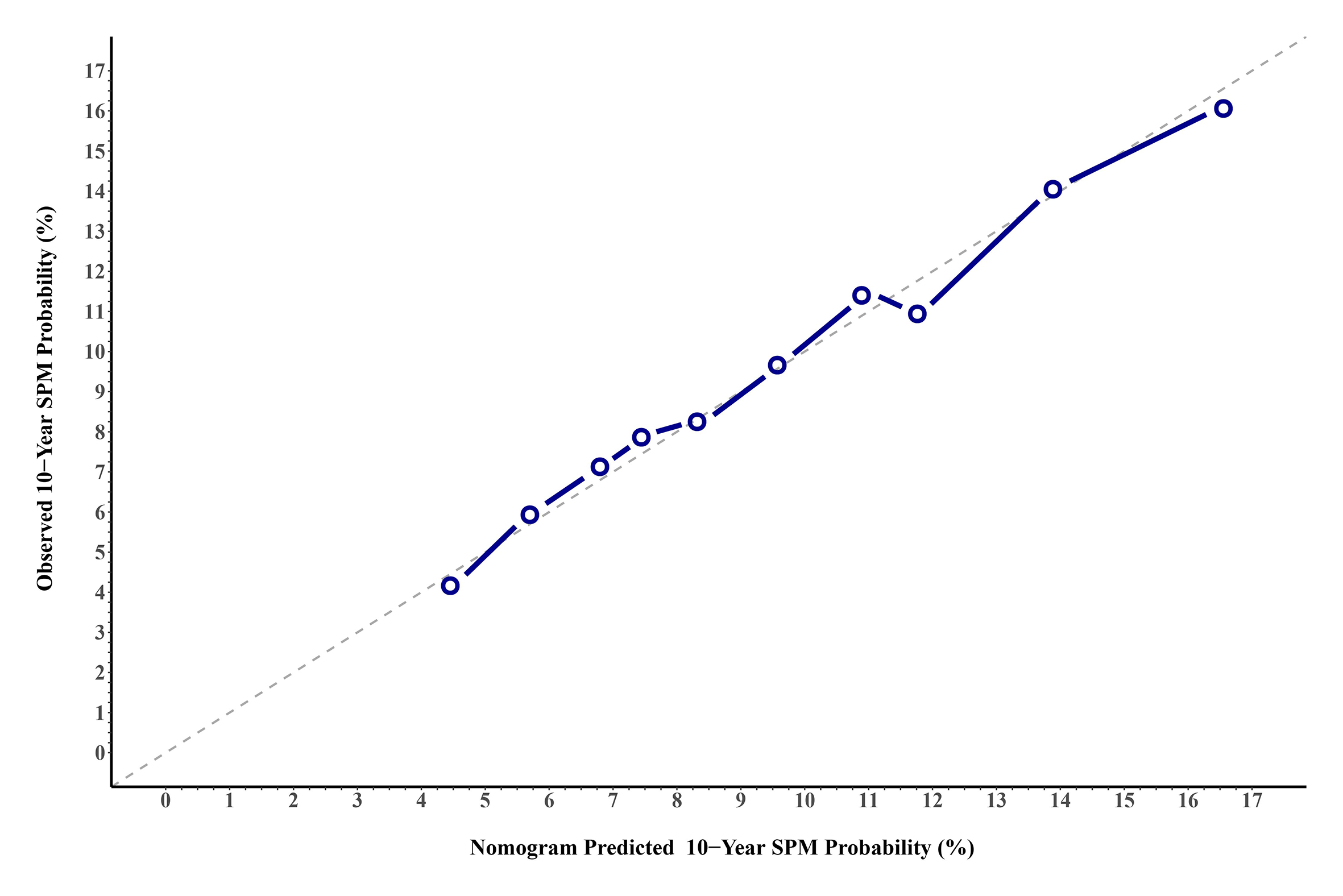

Supplement: Supplementary Figure 4 — Calibration curves for 10-year predicted vs. observed probability of developing second primary malignancies estimated by the Fine and Gray model. [file Image_4.tif]
