# Supplementary material for: Risk Prediction of Second Primary Malignancies in Primary Early-Stage Ovarian Cancer Survivors: A SEER-Based National Population-Based Cohort Study
Source: Front Oncol. 2022 May 19;12:875489. doi: 10.3389/fonc.2022.875489 (PMC9161780; doi:10.3389/fonc.2022.875489)
Supplement: Supplementary file 5 [file Table_1.docx]

**Supplementary Table 1.** The stages of ovarian cancer recoded according to 8^th^ edition of AJCC cancer staging manual. *

| AJCC 8^th^ stage | T stage | N satge | M stage |
| --- | --- | --- | --- |
| Stage I | T1 | N0 | M0 |
| Stage II | T2 | N0 | M0 |
| Stage III | T3 | N0 | M0 |
|  | T0, T1, T2, T3 | N1 | M0 |
| Stage IV | T4a  T0, T1, T2, T3, T4a Any T  T4b  Any T | N0, N1  N2  N3  Any N  Any N | M0  M0  M0  M0  M1 |

* The original stages of ovarian cancer in the SEER database were coded during year 1998-2003 and 2004-2013 according to the AJCC Cancer Staging Manual, Edition 3^rd^ and 6^th^, respectively.
