# Supplementary material for: Risk Prediction of Second Primary Malignancies in Primary Early-Stage Ovarian Cancer Survivors: A SEER-Based National Population-Based Cohort Study
Source: Front Oncol. 2022 May 19;12:875489. doi: 10.3389/fonc.2022.875489 (PMC9161780; doi:10.3389/fonc.2022.875489)
Supplement: Supplementary file 6 [file Table_2.docx]

**Supplementary Table 2.** Histology classification scheme of the ovarian cancer based on the ICD-O-3 morphology/behavior codes.*

| Histology | ICD-O-3 morphology/behavior codes |
| --- | --- |
| Non-epithelial | 8240/3, 8243/3, 8244/3, 8245/3, 8330/3, 8331/3, 8335/3, 8340/3, 8590/3, 8593/3, 8600/3, 8620/3, 8621/3, 8622/3, 8623/3, 8631/3, 8634/3, 8640/3, 8650/3, 8670/3, 8720/3, 8800/3, 8801/3, 8802/3, 8804/3, 8805/3, 8806/3, 8810/3, 8815/3, 8830/3, 8851/3, 8854/3, 8890/3, 8891/3, 8896/3, 8900/3, 8901/3, 8902/3, 8910/3, 8920/3, 8936/3, 8990/3, 9044/3, 9120/3, 9220/3, 9260/3, 9391/3, 9473/3, 9500/3, 9580/3, 9060/3, 9064/3, 9065/3, 9070/3, 9071/3, 9073/3, 9080/3, 9081/3, 9082/3, 9083/3, 9084/3, 9085/3, 9090/3, 9091/3, 9100/3, 9101/3, 9105/3, 9180/3, 9364/3, 9400/3, 9460/3, 9580/3 |
| Serous | 8020/3, 8021/3, 8022/3, 8050/3, 8120/3, 8130/3, 8260/3, 8441/3, 8442/3, 8450/3, 8460/3, 8461/3, 8462/3, 8463/3, 9014/3 |
| Endometrioid | 8380/3, 8381/3, 8382/3, 8383/3, 8482/3, 8560/3, 8570/3, 8930/3, 8931/3 |
| Mucinous | 8470/3, 8471/3, 8472/3, 8480/3, 8481/3, 8482/3, 8490/3, 9015/3 |
| Clear cell | 8005/3, 8084/3, 8290/3, 8310/3, 8313/3, 8443/3, 8444/3, 9110/3 |
| Brenner carcinoma | 8010/3, 8046/3, 8140/3, 8230/3, 8440/3, 9000/3 |
| Other epithelial** | 8011/3, 8012/3, 8013/3, 8030/3, 8031/3, 8032/3, 8033/3, 8040/3, 8041/3, 8043/3, 8045/3, 8052/3, 8070/3, 8071/3, 8072/3, 8073/3, 8074/3, 8075/3, 8082/3, 8123/3, 8141/3, 8144/3, 8200/3, 8246/3, 8249/3, 8250/3, 8255/3, 8262/3, 8263/3, 8312/3, 8320/3, 8323/3, 8337/3, 8341/3, 8344/3, 8384/3, 8401/3, 8410/3, 8430/3, 8452/3, 8500/3, 8504/3, 8507/3, 8542/3, 8562/3, 8574/3, 8575/3, 8576/3, 8503/3, 8933/3, 8935/3, 8940/3, 8963/3, 8250/3, 8255/3, 8323/3, 8950/3, 8951/3, 8960/3, 8980/3, 8981/3 |

* The histologic types of ovarian cancer were classified according to WHO histologic classification and relevant literature, as well as considering the actual sample size of each histologic type in our dataset.

** Other epithelial histology including carcinosarcoma, large cell, giant cell, spindle cell, pseudo sarcomatous, mixed histological types and so on.
