# Supplementary material for: Risk Prediction of Second Primary Malignancies in Primary Early-Stage Ovarian Cancer Survivors: A SEER-Based National Population-Based Cohort Study
Source: Front Oncol. 2022 May 19;12:875489. doi: 10.3389/fonc.2022.875489 (PMC9161780; doi:10.3389/fonc.2022.875489)
Supplement: Supplementary file 7 [file Table_3.docx]

**Supplementary Table 3.** Detailed point of each value for the risk predictors and of each risk group stratified by competing-risk nomogram.

| Subgroup | Point |
| --- | --- |
| Age at initial diagnosis, in years |  |
| 18-49 | 0.0 |
| 50-64 | 60.1 |
| 65-79 | 100.0 |
| Race |  |
| Asian pacific | 6.3 |
| White | 35.7 |
| Black | 44.4 |
| Other | 0.0 |
| Histology |  |
| Non-epithelial | 0.0 |
| Serous | 51.5 |
| Endometrioid | 57.7 |
| Mucinous | 59.1 |
| Clear cell | 16.9 |
| Brenner tumor | 47.6 |
| Other epithelial | 38.2 |
| No. of lymph nodes examined |  |
| ≥12 | 0.0 |
| <12 | 24.6 |
| Radiotherapy |  |
| None | 0.0 |
| Yes | 86.9 |
| Nomogram-based stratification ^a^ |  |
| Low-risk group | 0.0-98.5 |
| Intermediate-risk group | 98.6-178.1 |
| High-risk group | 178.2-315.0 |

^a^ The stratification was determined according to the total points calculated by the nomogram.
